# Ground Target Tracking Using an Airborne Angle-Only Sensor with Terrain Uncertainty and Sensor Biases

**DOI:** 10.3390/s22020509

**Published:** 2022-01-10

**Authors:** Dipayan Mitra, Aranee Balachandran, Ratnasingham Tharmarasa

**Affiliations:** Department of Electrical & Computer Engineering, McMaster University, Hamilton, ON L8S 4K1, Canada; a16@mcmaster.ca (A.B.); thamas@mcmaster.ca (R.T.)

**Keywords:** angle-only sensor, terrain uncertainty, posterior Cramer–Rao lower bound, bias estimation, path planning

## Abstract

Airborne angle-only sensors can be used to track stationary or mobile ground targets. In order to make the problem observable in 3-dimensions (3-D), the height of the target (i.e., the height of the terrain) from the sea-level is needed to be known. In most of the existing works, the terrain height is assumed to be known accurately. However, the terrain height is usually obtained from Digital Terrain Elevation Data (DTED), which has different resolution levels. Ignoring the terrain height uncertainty in a tracking algorithm will lead to a bias in the estimated states. In addition to the terrain uncertainty, another common source of uncertainty in angle-only sensors is the sensor biases. Both these uncertainties must be handled properly to obtain better tracking accuracy. In this paper, we propose algorithms to estimate the sensor biases with the target(s) of opportunity and algorithms to track targets with terrain and sensor bias uncertainties. Sensor bias uncertainties can be reduced by estimating the biases using the measurements from the target(s) of opportunity with known horizontal positions. This step can be an optional step in an angle-only tracking problem. In this work, we have proposed algorithms to pick optimal targets of opportunity to obtain better bias estimation and algorithms to estimate the biases with the selected target(s) of opportunity. Finally, we provide a filtering framework to track the targets with terrain and bias uncertainties. The Posterior Cramer–Rao Lower Bound (PCRLB), which provides the lower bound on achievable estimation error, is derived for the single target filtering with an angle-only sensor with terrain uncertainty and measurement biases. The effectiveness of the proposed algorithms is verified by Monte Carlo simulations. The simulation results show that sensor biases can be estimated accurately using the target(s) of opportunity and the tracking accuracies of the targets can be improved significantly using the proposed algorithms when the terrain and bias uncertainties are present.

## 1. Introduction

Tracking a ground target using an airborne sensor platform is frequently used in various applications, such as surveillance, search, and rescue missions [1,2,3]. Airborne radar sensors are of particular interest in various surveillance missions, because of their ‘day-and-night’ operational capabilities [4]. Airborne synthetic aperture radars (SAR) are often used to acquire high-resolution images of ground targets [5,6]. In [7,8], authors presented the application of airborne sensors for magnetic anomaly detection (MAD). MAD is widely used in maritime surveillance, detection of shipwrecks, geophysical studies, etc. [7].

Estimating the state of a ground target using the measurements from an angle-only airborne sensor is one of the most practical applications. A number of works have extensively studied the angle-only tracking problem in the 2-D Cartesian Coordinate System (CCS) [9,10]. However, as the authors in [2] pointed out, the number of works on 3-D angle-only tracking problems is relatively low. Some of the earlier works involving tracking ground targets in the 3-D coordinate system using angle-only sensors are reported in [11,12]. In [11], tracking an air target using a ground-based angle-only sensor is considered. Tracking a ground target using an airborne angle-only sensor is a slightly different problem since additional information about the target height will be available. In this work, our focus is to track a ground target using an airborne angle-only sensor platform.

One of the major challenges in angle-only tracking is observability. Considering the additional information of the height of the target from the sea level, such observability issue can be addressed. Since the target is on the ground, the target’s height is the same as the height of the terrain from the sea level, which can be obtained from pre-stored Digital Terrain Elevation Data (DTED) [13]. Most of the aforementioned works in 3-D angle-only tracking considered the height of the ground target from the sea level is known accurately [14,15,16]. However, in practical setups, such height information is associated with uncertainty due to the errors in the DTED data. Ignoring the height uncertainty (i.e., using a wrong height value) will lead to a bias in the estimated state. To the best of our knowledge, not many analysis is performed on tracking a ground target with terrain uncertainty. This is the motivation for this work to develop algorithms to handle the terrain uncertainty with angle-only sensors.

Apart from the terrain uncertainty, possible biases in the sensor measurements play a key role in determining the quality of the estimates. Possible sources of bias include sensor alignment bias, sensor altitude bias, location bias, etc. [17]. In this work, we consider only the measurement biases, i.e., the biases in the elevation and bearing angles. Sensor biases and terrain uncertainty should be handled jointly in order to obtain better tracking results.

In this work, we propose a filtering algorithm to track a target with bias and altitude uncertainties. With a larger bias uncertainty, a filter will take a longer time to reduce the target state bias. If we have an option to reduce the sensor bias uncertainty before we start tracking the target of interest, that will help to obtain a better estimate of the target faster. Usually, an airborne platform will fly from a base station to the Region Of Interest (ROI), where we have the target of interest. On the way to the ROI, a bias estimation could be performed by pointing the angle-only sensor toward one more multiple stationary ground object, called as targets of opportunity. In this work, this bias estimation is considered an optional step. Note that the bias uncertainty will not be completely removed even with this optional step. Hence, the filtering algorithm used for tracking the target should still consider the bias uncertainty.

In this work, we explore the possibility of improving the bias estimation using targets of opportunity by changing sensor trajectory. We also study the effect of increasing the number of targets of opportunity and changing their locations with respect to the sensor trajectory on bias estimation. Our proposed approach considers the bias estimation when the x and y coordinates of the target of opportunity are known as well as unknown. A number of bias estimation approaches are proposed in the literature [18,19,20]. However, the challenges in the bias estimation with the terrain uncertainty are not considered in any of the papers.

Predicting the performance of an estimator is essential to decide the optimal sensor trajectory or optimal targets of opportunity locations. The covariance of an unbiased estimator is bounded by the Posterior Cramer–Rao Lower Bound (PCRLB) [21,22]. When the estimator is biased, the estimated covariance can not be directly bounded by the PCRLB. In [23], the authors proposed a performance bound considering the gradients of the bias state. Such a performance bound on the total variance of the estimator is referred to as *biased PCRLB* [24]. The central idea behind using the gradient of the bias state is to have a dependency on the non-constant part of the bias. In other words, the bias can not be removed from the measurements by simple subtraction. In this work, PCRLB and a biased PCRLB are derived for angle-only tracking problems with bias and terrain uncertainties.

In this paper, we consider two possible scenarios: ground target with terrain uncertainty (1) remains stationary; (2) moves with a nearly constant velocity (CV) model [25]. We assume that the ground target (while moving in nearly CV) moves along the x–y plane, i.e., there is no velocity along the z-axis. Although the dynamic model of the target state is linear, the angle-only measurements are non-linear functions of the target and the sensor state [11]. For such an estimation problem, several non-linear filtering algorithms are proposed in the literature. Some of the examples include Extended Kalman Filter (EKF) [26], Cubature Kalman filter (CKF) [27], Unscented Kalman Filter (UKF) [28], and Feedback Particle Filter (FPF) [29].

The computational complexity of the UKF is of the same order as the EKF while providing improved estimation accuracy addressing the approximation issues of the EKF, as shown in [30,31]. As a result, we propose a filtering algorithm using UKF. However, UKF can easily be replaced by any other non-linear filter. One of the challenges in the ground target tracking problem is filter initialization with terrain uncertainty. In this work, we use the measurements obtained by the biased angle-only sensor to initialize the target state by incorporating the terrain uncertainty. The estimate errors of the target trajectory are compared with the PCRLB and a conventional approach to evaluate the accuracy and the benefit of the proposed algorithms. The simulation results show that the proposed approach provides better tracking results with all the given uncertainties.

The key contributions of our work can be stated as follows: (1) We derive the PCRLB for this problem to predict/evaluate the performance of the estimator and optimize bias estimation; (2) Bias estimation using a separate target(s) of opportunity is proposed with optimal platform trajectory and optimal target of opportunity location selection; (3) We propose a filtering approach to estimate a target with bias and terrain uncertainties.

This paper is organized as follows. We discuss the problem description in Section 2. System model detailing the coordinate system, measurement generation, and the system dynamics are introduced in Section 3. A discussion on performance bounds is presented in Section 4. Bias estimation approaches and related analysis are detailed in Section 5. Filter initialization, optional bias compensation, and ground target tracking are discussed in Section 6. Simulation results are shown in Section 7 and the paper ends with the concluding remarks in Section 8.

## 2. Problem Description

In this paper, our main objective is to track a ground target in 3-D using a biased airborne angle-only sensor. The ground target can either remain stationary or move at a nearly constant velocity. The height of the ground target from the sea level is obtained from DTED; hence it has uncertainty. The two major sources of uncertainty are measurement bias and terrain uncertainty.

To reduce the measurement bias uncertainty, the possible biases could be estimated using separate target(s) of opportunity on the way to the region of interest from the base station. Two possible cases that can happen with the target(s) of opportunity are (1) *x* and *y* coordinates of the target(s) are known accurately, but the *z* coordinate is obtained from the DTED data, which has error; (2) *x* and *y* coordinates of the target(s) are unknown, but the *z* coordinate is obtained from the DTED data as in the first case. Platform trajectory and the location of the target(s) of opportunity can be optimized to obtain a better bias estimate by minimizing the additional time required to reach the destination. That is, we prefer if we do not need to change the trajectory of the platform.

In this work, we make the following assumptions:

- Only a single target is considered. However, the proposed algorithm can be used for multiple well-separated targets without any modification;
- The height of the ground target from the sea level is fixed, but not known accurately;
- The ground target can either remain stationary or move with a nearly constant velocity;
- Bias affecting the angle-only measurements are unknown constant and additive. Time-varying bias is not considered in this work. However, the proposed approach can be easily extended for time-varying biases;
- Data association issues are not considered, i.e., false alarms are not considered.

In the next section, we introduce the coordinate system, the system dynamics and the measurement model.

## 3. System Model

### 3.1. Coordinate System

The states of the ground target and the own-ship at time step *k* are defined as $x_{k}^{t}={\left[x_{k}^{t},{\dot{x}}_{k}^{t},y_{k}^{t},{\dot{y}}_{k}^{t},z_{k}^{t}\right]}^{T}$ and $x_{k}^{o}={\left[x_{k}^{o},{\dot{x}}_{k}^{o},y_{k}^{o},{\dot{y}}_{k}^{o},z_{k}^{o}\right]}^{T}$, respectively, where ${\left(.\right)}^{T}$ denote the transpose operation. Note that the state of a stationary target is expressed as $x^{t}={\left[x^{t},y^{t},z^{t}\right]}^{T}$. Here, (x,y,z) represent the three axes of 3-D CCS and the superscripts ‘*t*’ and ‘*o*’ are reserved to denote the target and the own-ship (airborne sensor platform), respectively. In this manuscript, the terms own-ship and airborne sensor platform have been used interchangeably. The velocity is denoted as [$\dot{x}$, $\dot{y}$]. Note that there is no velocity across *z*-axis for the target and the own-ship, i.e., ${\dot{z}}_{k}^{t}=0$ and ${\dot{z}}_{k}^{o}=0$. It is assumed that the ground is flat through out the region where the target moves, hence the target’s *z* does not change over time, i.e., $z_{k}^{t}=z^{t}$.

In this work, we considered a locally flat earth model for all our calculations.

### 3.2. System Dynamics

In this work, a constant velocity (CV) model is used to model the system dynamics of the ground target. Given the discrete-time state of a moving target $x_{k}^{t}$, the state evolution is expressed as,

(1) $$x_{k+1}^{t}=F_{k}x_{k}^{t}+G_{k}v_{k}$$

where $F_{k}$ and $G_{k}$ are the state transition and the gain matrices, respectively. The process noise $v_{k}$ is a zero-mean Gaussian with covariance $Q_{k}$, i.e., $v_{k}∼N\left(v_{k};0,Q_{k}\right)$. For a stationary ground target, $F_{k}$ is an identity matrix and the noise part is zero, hence $x_{k+1}^{t}=x_{k}^{t}$.

Motion legs with CV and Constant Turn (CT) are used to model the system dynamics of the own-ship trajectory. Transition matrices of CV and CT models are given in (A1) and (A2), respectively.

### 3.3. Measurement Model

**Figure 1:**
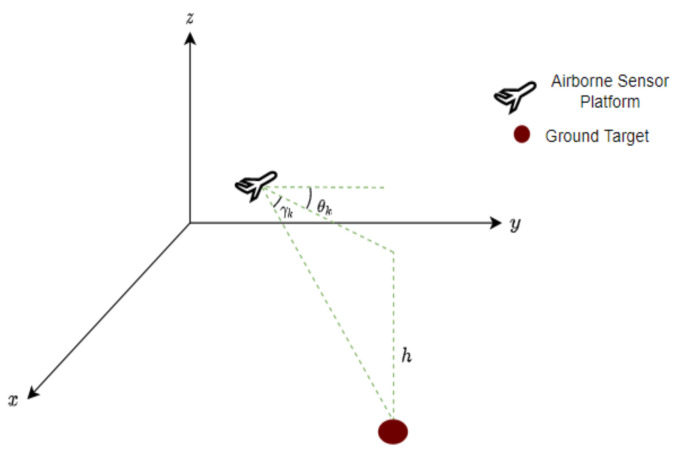
Target-sensor geometry.

Figure 1 shows the target-sensor geometry in 3-D CCS. The on-board angle-only sensor provides the bearing $\theta_{k}\in \left[-\pi ,\pi \right]$ and elevation $\gamma_{k}\in \left[-\frac{\pi }{2},\frac{\pi }{2}\right]$ measurements. As the own-ship trajectory is deterministic, $z_{k}^{o}$ is known for all time-steps.

Based on the assumptions from Section 2, a measurement is available for the target height from the sea level, $z_{m}$,

(2) $$z_{m}=z^{t}+v^{z}$$

where $v^{z}$ is a Gaussian noise with zero-mean and standard deviation $\sigma_{z^{t}}$, i.e., $v^{z}\;∼\;N\left(v^{z};0,\sigma_{z^{t}}^{2}\right)$.

The expressions for the true bearing and elevation angles are given by,

(3) $$\begin{array}{ccc} \theta_{k}^{\mathrm{true}} & = & \tan^{-1}\left[\left(x_{k}^{t}-x_{k}^{o}\right),\left(y_{k}^{t}-y_{k}^{o}\right)\right] \end{array}$$

(4) $$\begin{array}{ccc} \gamma_{k}^{\mathrm{true}} & = & \tan^{-1}\left[\left(z_{k}^{t}-z_{k}^{o}\right),\sqrt{{\left(x_{k}^{t}-x_{k}^{o}\right)}^{2}+{\left(y_{k}^{t}-y_{k}^{o}\right)}^{2}}\right] \end{array}$$

Let us denote the bias vector of the sensor as $b_{k}={\left[\theta_{b_{k}},\gamma_{b_{k}}\right]}^{T}$. In this work, we consider the measurement biases to be additive and unknown constants. As a result, a separate state transition matrix for the bias state evolution is not necessary. Measurement model for acquiring bearing and elevation from the angle-only sensor is expressed as,

(5) $$z_{k}=h\left(x_{k}^{t},x_{k}^{o}\right)+b_{k}+w_{k}$$

where $h\left(x_{k}^{t},x_{k}^{o}\right)={\left[\theta_{k}^{\mathrm{true}},\gamma_{k}^{\mathrm{true}}\right]}^{T}$ and measurement noise $w_{k}∼N\left(w_{k};0,R_{k}\right)$ with covariance $R_{k}$ = diag$\left(\sigma_{\theta }^{2},\sigma_{\gamma }^{2}\right)$. Here, $\sigma_{\theta }$ and $\sigma_{\gamma }$ are the standard deviations for the bearing and the elevation measurements, respectively.

In the next section, we introduce the necessary bounds to evaluate the performance of an estimator.

## 4. Performance Bound

### 4.1. Posterior Cramer–Rao Lower Bound

Let us define $Z_{K}$ as the collection of all the measurements for time k=1,…,K, i.e., $Z_{K}=\left[z_{1},z_{2},\ldots ,z_{K}\right]$. The objective of an estimator is to find the conditional probability distribution $p(x_{k}^{t}|Z_{K})$. Associated covariance matrix $\left(C_{{\hat{x}}^{t}}\right)$ of an unbiased estimate of the target state $x_{k}^{t}$ can be lower bounded by,

(6) $$\begin{array}{c} C_{{\hat{x}}^{t}}=E\left[\left({\hat{x}}_{k}^{t}-x_{k}^{t}\right){\left({\hat{x}}_{k}^{t}-x_{k}^{t}\right)}^{T}\right]\geq J_{k}^{-1} \end{array}$$

where $J_{k}$ is the Fisher Information Matrix (FIM) [21]. The above bound is called the PCRLB. In [32] Tichavsky et al. proposed a recursive formulation to obtain the FIM. In our problem, the target state evaluation is modeled by a discrete-time linear system. Knowing the state transition matrix $F_{k}$, the process noise covariance $Q_{k}$ and the measurement noise covariance $R_{k}$, a simplified expression for the recursive formulation of the FIM is expressed as,

(7) $$\begin{array}{c} J_{k+1}={\left(F_{k}J_{k}^{-1}F_{k}^{T}+Q_{k}\right)}^{-1}+J_{z}\left(k+1\right) \end{array}$$

where $J_{z}\left(k+1\right)$ is called the measurement contribution to the FIM, which is evaluated as

(8) $$\begin{array}{c} J_{z}\left(k\right)=E\left[q_{k}H_{k}^{T}R_{k}^{-1}H_{k}\right] \end{array}$$

Here $H_{k}$ is the Hessian matrix of the measurement equation (see (A3) for details on the matrix construction) and $q_{k}$ is called Information Reduction Factor (IRF). In this work, we do not consider the possibility of false alarms or miss detection. Hence, data association uncertainty is absent, i.e., IRF $q_{k}=1$.

Finally, substituting $J_{z}\left(k+1\right)$ from (8) into (7), the FIM is evaluated as,

(9) $$\begin{array}{c} J_{k+1}={\left(F_{k}J_{k}^{-1}F_{k}^{T}+Q_{k}\right)}^{-1}+H_{k}^{T}R_{k}^{-1}H_{k} \end{array}$$

Assuming the initial target state covariance to be $P_{1}$, FIM is initialised as $J_{1}=P_{1}^{-1}$.

### 4.2. Presence of Measurement Bias

It is well known that satisfying certain regulatory conditions, the performance of an unbiased estimator is bounded by the PCRLB. However, in this problem, we consider the additional uncertainty caused by the measurement bias. Hence, the estimates obtained by the biased angle-only measurements can not be bounded directly by the PCRLB without accounting for the additional bias uncertainty in the FIM (shown in (9)). In this section, we discuss the following modifications to the FIM for obtaining a suitable performance bound for this work.

Recalling the governing equation of $J_{z}\left(k\right)$, it is clear that the measurement bias is going to impact the measurement noise covariance $R_{k}$, whereas the Hessian matrix remains unchanged. First, we form the covariance matrix for the bias as $R_{\mathrm{bias}}=\mathrm{diag}\left(\sigma_{\theta }^{2},\sigma_{\gamma }^{2}\right)$. To provide bias compensation we obtain the bias compensated measurement covariance matrix as,

(10)Rkb=Rk+Rbias(11)      =σθ2+σθb200σγ2+σγb2

Once the bias compensated measurement covariance matrix is obtained, substituting $R_{k}^{b}$ into $R_{k}$ of (9) we obtain,

(12) $$\begin{array}{c} J_{k+1}={\left(F_{k}J_{k}^{-1}F_{k}^{T}+Q_{k}\right)}^{-1}+H_{k}^{T}{\left(R_{k}^{b}\right)}^{-1}H_{k} \end{array}$$

Now, we focus on modifying the FIM to account for the terrain uncertainty.

### 4.3. Presence of Terrain Uncertainty

The prior information about the terrain height (2) is used to initialize the FIM at k=1. Note that this measurement can not be used more than once. In order to initialize the FIM at k=1 using the terrain height information and the first target measurement, we consider a stacked measurement $\left[\theta_{k},\gamma_{k},z_{m}\right]$ and corresponding covariance matrix $R_{z}=\mathrm{diag}\left(\sigma_{\theta }^{2},\sigma_{\gamma }^{2},\sigma_{z^{t}}^{2}\right)$. The subscript ‘*z*’ indicates the initial time step where the terrain uncertainty is considered. We use this notation throughout this paper. Combining with $R_{\mathrm{bias}}$ the bias compensated covariance matrix is expressed as,

(13)Rzb=Rz+Rbias(14)     =σθ2+σθb2000σγ2+σγb2000σzt2

Once $R_{z}^{b}$ is formed, corresponding Hessian matrix $H_{z}$ is evaluated as shown in (A4). Using $R_{z}^{b}$ and the newly evaluated $H_{z}$, we can initialize FIM at k=1 as

(15) $$\begin{array}{c} J_{1}={\left(F_{k}J_{k}^{-1}F_{k}^{T}+Q_{k}\right)}^{-1}+{\left(H_{z}\right)}^{T}{\left(R_{z}^{b}\right)}^{-1}H_{z} \end{array}$$

### 4.4. Biased Posterior Cramer–Rao Lower Bound

A bound on the covariance of biased target state estimate ${\hat{x}}_{k}^{t}$ is defined as biased PCRLB. In [21,23,24], the biased PCRLB is expressed as,

(16) $$\begin{array}{ccc} C_{{\hat{x}}^{t}} & \geq & {\left(I+D_{k}\right)}^{T}J_{k}^{-1}\left(I+D_{k}\right) \end{array}$$

where $D_{k}$ is the bias gradient matrix. The FIM is evaluated following (9). The bias in the state is written as $b_{x_{k}^{t}}=\left(E\left[{\hat{x}}_{k}^{t}\right]-x_{k}^{t}\right)$. Please note that the bias in the state is denoted as $b_{x_{k}^{t}}$, which is not to be confused with the measurement bias b. Given the target state $x_{k}^{t}$, bias gradient matrix is defined as,

(17) $$\begin{array}{c} D_{k}=\frac{\partial b_{x_{k}^{t}}}{\partial x_{k}^{t}} \end{array}$$

In order to empirically obtain $D_{k}$ from (17), first we have to form the bias state. For a stationary ground target, the bias in the state vector is denoted as $b_{x_{k}^{t}}=\left[b_{x_{k}^{t}},b_{y_{k}^{t}},b_{z_{k}^{t}}\right]$. Analytical form of the individual bias state coordinates can be obtained as,

(18) $$\begin{array}{ccc} b_{x_{k}^{t}} & = & \frac{z_{k}^{t}\sin\left(\theta_{k}^{\mathrm{true}}+\theta_{b_{k}}\right)}{\tan(\gamma_{k}^{\mathrm{true}}+\gamma_{b_{k}})}-\frac{z_{k}^{t}\sin\left(\theta_{k}^{\mathrm{true}}\right)}{\tan(\gamma_{k}^{\mathrm{true}})} \end{array}$$

(19) $$\begin{array}{ccc} b_{y_{k}^{t}} & = & \frac{z_{k}^{t}\cos\left(\theta_{k}^{\mathrm{true}}+\theta_{b_{k}}\right)}{\tan(\gamma_{k}^{\mathrm{true}}+\gamma_{b_{k}})}-\frac{z_{k}^{t}\cos\left(\theta_{k}^{\mathrm{true}}\right)}{\tan(\gamma_{k}^{\mathrm{true}})} \end{array}$$

The bias gradient $D_{k}$ is evaluated as shown below,

(20) $$\begin{array}{ccc} D_{k} & = & \left[\begin{array}{ccc} \frac{\partial b_{x_{k}^{t}}}{\partial x_{k}^{t}} & \frac{\partial b_{x_{k}^{t}}}{\partial y_{k}^{t}} & \frac{\partial b_{x_{k}^{t}}}{\partial z_{k}^{t}}\\ \frac{\partial b_{y_{k}^{t}}}{\partial x_{k}^{t}} & \frac{\partial b_{y_{k}^{t}}}{\partial y_{k}^{t}} & \frac{\partial b_{y_{k}^{t}}}{\partial z_{k}^{t}}\\ \frac{\partial b_{z_{k}^{t}}}{\partial x_{k}^{t}} & \frac{\partial b_{z_{k}^{t}}}{\partial y_{k}^{t}} & \frac{\partial b_{z_{k}^{t}}}{\partial z_{k}^{t}} \end{array}\right] \end{array}$$

The derivatives of (20) can be evaluated easily and not shown here in this paper. Additionally, modifying (20) for moving ground target is straightforward and not shown in this paper.

Intuitively the bias gradient represents the part of the bias which can not be removed by simple subtraction, i.e., the non-additive component of the bias. As a result, the biased PCRLB, shown in (16), depends on the gradient matrix $D_{{}_{k}}$. Performance of the ground target tracking, both stationary and moving, is validated against both PCRLB and biased PCRLB in Section 7.2.

In the next section, we use the derived PCRLB to optimize the platform trajectory to estimate the sensor biases using targets of opportunity.

## 5. Bias Estimation Using Targets of Opportunity

Recalling the discussions from Section 2, an optional two-step bias estimation using targets of opportunity is presented in this section. To bring clarity and avoid confusion with the original target, throughout this paper, we reserve the term ‘target of opportunity’ to indicate the target for which some prior information is known, or we are not interested in estimating that target’s state. Note that this step is usually performed on the way to the target region from the base station. Hence, we need to consider only a reduced bias error when estimating the target state.

The two steps involved in our proposed bias estimation approach are as follows. The first step is to identify one or more stationary targets of opportunity on the sensor’s path to the tracking region. The next step is to estimate the biases in the measurements using the identified targets of opportunity. Such a bias estimation approach has two following benefits:

- The uncertainty in the sensor bias is reduced before the original ground target appears in the sensor’s field of view. Hence, the tracking can provide better estimates from the beginning. Otherwise, we may need to make more maneuvers to reduce the biases in the state estimate to obtain a reasonable tracking accuracy;
- The sensor can choose multiple targets of opportunity to improve the convergence of the bias estimates. When we use only the interested target to correct the bias, it will take a longer time to converge.

In this section, we analyze the impact on bias estimation caused by the change in sensor trajectory, the number of targets of opportunity used and targets’ proximity to the sensor trajectory. We consider both scenarios where the locations of the targets of opportunity are known as well as unknown, but the terrain heights are not known accurately. PCRLB is used to quantify the estimation quality in order to find the optimal platform trajectory and the locations of the targets of opportunity. The terms ‘target of opportunity’ and ‘target of opportunity with terrain uncertainty’ are used interchangeably in this section.

### 5.1. Known Location with Terrain Uncertainty

In this section, the bias estimation is performed with a known location of the target of opportunity. To emphasize, the *x* and *y* coordinates of the target of opportunity are known, and the height (*z*) information is obtained from DTED. Hence, there is an uncertainty in the target’s *z* value. The following factors impact the bias estimation: the number of targets of opportunity used, change in sensor trajectory and the proximity of the target of opportunity with the sensor trajectory. In this section, two possible scenarios are analyzed, and a conclusion on the optimal bias estimation is presented using the PCRLB. In the first scenario, the sensor bias is estimated using one target of opportunity with various sensor trajectories. In the second scenario, the sensor bias is estimated using two targets of opportunity with possibly different terrain heights.

Let us denote the state vector of the stationary target of opportunity as $x_{k}^{to}={\left[x_{k}^{to},y_{k}^{to},z_{k}^{to}\right]}^{T}$. Here, the superscript ‘to’ indicates the target of opportunity. The uncertainty in the height of the target of opportunity is modeled as a zero-mean Gaussian with a standard deviation of $\sigma_{z^{to}}$. Although the location of the target of opportunity is known, $z_{k}^{to}$ is needed to be estimated because of the presence of the terrain uncertainty. Therefore, the state vector of the bias estimation problem at the time step *k* is expressed as $x_{k}^{\mathrm{aug}1}={\left[z_{k}^{to},\theta_{b_{k}},\gamma_{b_{k}}\right]}^{T}$. Here, the post-fix ‘aug1’ refers to the augmented state for the first scenario, i.e., known location of the target of opportunity.

Our next step is to initialize the filter for bias estimation. Modeling the error associated with the terrain uncertainty by a Gaussian with zero-mean and $\sigma_{z^{to}}$ standard deviation, we can write $z_{m}^{to}∼N\left(z_{m}^{to};z^{to},\sigma_{z^{to}}^{2}\right)$. Hence, we can initialize the height of the target of opportunity with the DTED information $z_{m}^{to}$. The bias states are initialized as ${\left[0,0\right]}^{T}$. Therefore, the state vector is initialized as $x_{1}^{\mathrm{aug}1}={\left[z_{m}^{to},0,0\right]}^{T}$. Moreover, the initial state covariance $P_{1}^{\mathrm{aug}1}$ is calculated using the FIM evaluated at the initial time step as $P_{1}^{\mathrm{aug}1}={\left(J_{1}^{\mathrm{aug}1}\right)}^{-1}$. After filter initialization, the non-linear filter (UKF in our work) is used to estimate the augmented state. Note that, for initialization $H_{z}^{\mathrm{aug}1}$ is expressed as,

(21) $$\begin{array}{ccc} H_{z}^{\mathrm{aug}1} & = & \left[\begin{array}{ccc} \frac{\partial z_{k}^{to}}{\partial z_{k}^{to}} & \frac{\partial z_{k}^{to}}{\partial \theta_{b_{k}}} & \frac{\partial z_{k}^{to}}{\partial \gamma_{b_{k}}}\\ \frac{\partial \theta_{k}}{\partial z_{k}^{to}} & \frac{\partial \theta_{k}}{\partial \theta_{b_{k}}} & \frac{\partial \theta_{k}}{\partial \gamma_{b_{k}}}\\ \frac{\partial \gamma_{k}}{\partial z_{k}^{to}} & \frac{\partial \gamma_{k}}{\partial \theta_{b_{k}}} & \frac{\partial \gamma_{k}}{\partial \gamma_{b_{k}}} \end{array}\right]=\left[\begin{array}{ccc} 1 & 0 & 0\\ 0 & 1 & 0\\ \frac{\sqrt{x_{k}^{{rto}^{2}}+y_{k}^{{rto}^{2}}}}{x_{k}^{{rto}^{2}}+y_{k}^{{rto}^{2}}+z_{k}^{{rto}^{2}}} & 0 & 1 \end{array}\right] \end{array}$$

The biased measurement covariance matrix, during initialization, of the augmented state is expressed as $R_{z}^{\mathrm{aug}1}=\mathrm{diag}\left(\sigma_{\theta }^{2},\sigma_{\gamma }^{2},\sigma_{z^{to}}^{2}\right)$. Once $H_{z}^{\mathrm{aug}1}$ and $R_{z}^{\mathrm{aug}1}$ are obtained for k=1, the FIM is evaluated from (9) as,

(22) $$\begin{array}{c} J_{1}^{\mathrm{aug}1}={\left(F_{k}J_{k}^{-1}F_{k}^{T}+Q_{k}\right)}^{-1}+{\left(H_{z}^{\mathrm{aug}1}\right)}^{T}{\left(R_{z}^{\mathrm{aug}1}\right)}^{-1}H_{z}^{\mathrm{aug}1} \end{array}$$

After initialization, we obtain the PCRLB for the time steps k>1. First, we obtain the Hessian matrix $H_{k}^{\mathrm{aug}1}$ of the bearing and elevation measurement model as,

(23) $$\begin{array}{ccc} H_{k}^{\mathrm{aug}1} & = & \left[\begin{array}{ccc} \frac{\partial \theta_{k}}{\partial z_{k}^{to}} & \frac{\partial \theta_{k}}{\partial \theta_{b_{k}}} & \frac{\partial \theta_{k}}{\partial \gamma_{b_{k}}}\\ \frac{\partial \gamma_{k}}{\partial z_{k}^{to}} & \frac{\partial \gamma_{k}}{\partial \theta_{b_{k}}} & \frac{\partial \gamma_{k}}{\partial \gamma_{b_{k}}} \end{array}\right]=\left[\begin{array}{ccc} 0 & 1 & 0\\ \frac{\sqrt{x_{k}^{{rto}^{2}}+y_{k}^{{rto}^{2}}}}{x_{k}^{{rto}^{2}}+y_{k}^{{rto}^{2}}+z_{k}^{{rto}^{2}}} & 0 & 1 \end{array}\right] \end{array}$$

where the relative state $x_{k}^{rto}=x_{k}^{to}-x_{k}^{o}$. The measurement contribution $J_{z}^{\mathrm{aug}1}\left(k\right)=\left(H_{k}^{\mathrm{aug}1}\right)$${\left(R_{k}^{\mathrm{aug}1}\right)}^{-1}H_{k}^{\mathrm{aug}1}$, where measurement noise covariance is $R_{k}^{\mathrm{aug}1}=\mathrm{diag}\left(\sigma_{\theta }^{2},\sigma_{\gamma }^{2}\right)$. With $J_{z}^{\mathrm{aug}1}\left(k\right)$, $H_{k}^{\mathrm{aug}1}$ and $R_{k}^{\mathrm{aug}1}$, PCRLB is evaluated using (9). As the sensor bias and the target height are constants, we consider $F_{k}=I$, i.e., identity matrix of appropriate dimension.

In order to improve the bias estimation further, we consider two different cases below.

#### 5.1.1. Change in Sensor Trajectory

**Figure 2:**
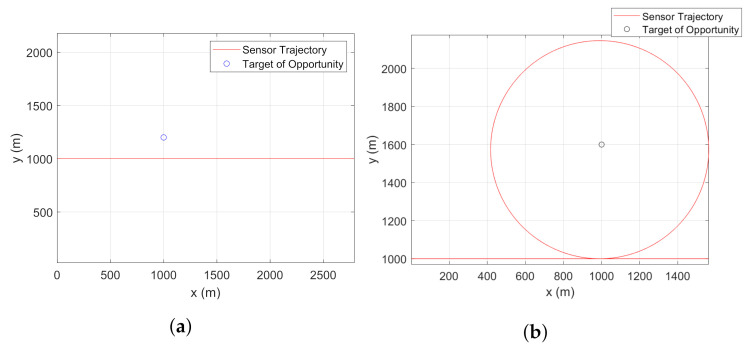
Sensor trajectories for bias estimation using a stationary target with known location and terrain uncertainty. Sensor trajectory follows: (**a**) the CV model, (**b**) the CV-CT model.

For a given target of opportunity, we restrict our observations to two types of sensor trajectories. The sensor can either follow the CV model and make a fly-by while estimating the bias state or follow a combination of CV and CT models to make a turn around the target to estimate the bias. In this work, the combination of CV and CT models is referred to as CV-CT model. The aforementioned two types of sample sensor trajectories are shown in Figure 2a,b, respectively.

Let us assume that the target of opportunity remains in the sensor’s field of view for *K* time steps. The *x*-axis indicates the sensor heading at the start time step. In other words, when the sensor follows the CV model, ${\dot{y}}_{k}^{o}=0$.

Let us now consider the case when the sensor follows the CV-CT model. We denote $K_{CV}$ as the total number of time steps the sensor follows the CV model in this CV-CT model. The platform switch to the CT model when the platform reaches the closest distance from the target of opportunity. Considering the sensor velocity to be *V*m/s and the sampling rate to be *T*, we can write the total number of samples obtained while the sensor remains in the CV model to be $\left(\frac{1}{T}\frac{|x_{1}^{to}-x_{1}^{o}|}{V}\right)$. Once the sensor starts following the CT model, the total number of measurements obtained by completing one full cycle is denoted by $\left(\frac{1}{T}\frac{2\pi }{\omega }\right)$, where ω is the turn rate. We consider the total number of time steps needed to obtain $\left(\frac{1}{T}\frac{2\pi }{\omega }\right)$ samples to be $K_{CT}$.

Now we shift our focus into finding ω, for the sensor to follow the CT model. Let us denote the x–y coordinates of the sensor at $k=K_{CV}$ as $\left(x_{K_{CV}}^{o},y_{K_{CV}}^{o}\right)$. From the discussions of the previous paragraph, the maximum change in the y-coordinate occurs at $k=\frac{K_{CT}}{2}$. In this work, the idea behind finding ω is to ensure that the target of opportunity is inside the circle formed by the CT model,

(24) $$\begin{array}{c} \left|y_{\frac{K_{CT}}{2}}^{o}-y_{K_{CV}}^{o}\right|\;\geq \;\left|y_{K_{CV}}^{o}-y_{1}^{to}\right| \end{array}$$

Now, using the CT model (A1), we can show that

(25) $$y_{\frac{K_{CT}}{2}}^{o}-y_{K_{CV}}^{o}=\frac{2}{\omega }{\dot{x}}_{K_{CV}}^{o}$$

From (25) and (24), we can obtain,

(26) $$\begin{array}{ccc} \frac{2}{\omega }{\dot{x}}_{K_{CV}}^{o} & \geq & \left|y_{K_{CV}}^{o}-y_{1}^{to}\right|\\ \omega & \leq & \frac{2}{\left|y_{K_{CV}}^{o}-y_{1}^{to}\right|}{\dot{x}}_{K_{CV}}^{o} \end{array}$$

However, the platform has a constraint on maximum turn, $\omega_{max}$, that it can make, hence we pick the ω as $\min\left(\omega_{max},\frac{2}{\left|y_{K_{CV}}^{o}-y_{1}^{to}\right|}{\dot{x}}_{K_{CV}}^{o}\right)$ to reduce the additional time required to reach the region of original target.

From (9), it is evident that the Hessian matrix $H_{k}^{\mathrm{aug}1}$ significantly affects the PCRLB. When considering (23), the elements corresponding to the differentiation involving $\theta_{k}$ in $H_{k}^{\mathrm{aug}1}$ are constant. Therefore, the measurement contribution from the bearing bias does not depend on the sensor trajectory. On the other hand, the terms corresponding to the differentiation involving $\gamma_{k}$ in $H_{k}^{\mathrm{aug}1}$ depends on the location of both the sensor and the target of opportunity. As a sensor trajectory formed by the CV-CT model reduces the relative distance between the target of opportunity and the sensor, $x_{k}^{rto}$ and $y_{k}^{rto}$ reduces. As a result, reduction in $\left(\frac{\sqrt{x_{k}^{{rto}^{2}}+y_{k}^{{rto}^{2}}}}{x_{k}^{{rto}^{2}}+y_{k}^{{rto}^{2}}+z_{k}^{{rto}^{2}}}\right)$ (from (23)) leads to the reduction in PCRLB. Thus, the sensor trajectory formed by the CV-CT model provides a better elevation bias estimation when compared to that of the sensor trajectory formed by the CV model. A comparative analysis between bias estimation performance while the sensor follows both the CV and the CV-CT model is shown in Section 7.3.1. Note that we need to spend more time on this bias estimation when we use the CV-CT model. With the CV model, no additional time is needed to scan the target of opportunity since there is no change in the platform trajectory.

We now expand our analysis to show the effect of using two targets of opportunity with known locations and additional terrain uncertainty.

#### 5.1.2. Bias Estimation with Multiple Targets of Opportunity

**Figure 3:**
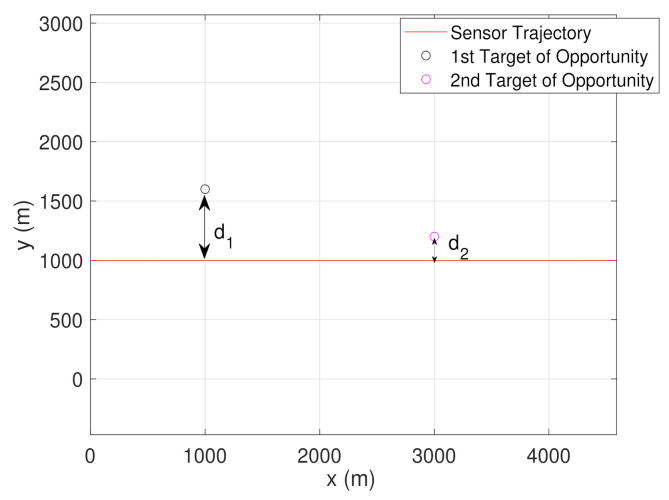
Sensor trajectory following CV model for bias estimation using two targets with known locations and terrain uncertainty.

In the previous section, we concluded that the sensor following a CV-CT trajectory improves the estimate ${\hat{\gamma }}_{b_{k}}$, when compared to the case where the sensor follows only a CV trajectory. In this section, we analyze the significance of adding a second stationary target of opportunity in the sensor’s field of view. The goal here is to analyze whether the presence of the second target of opportunity coupled with the sensor following the CV model provides better ${\hat{\gamma }}_{b_{k}}$ so that we do not need to change the platform trajectory. Note that the second target of opportunity with a different terrain height could be located anywhere in the sensor’s field of view. Figure 3 shows an example of two targets of opportunity located at a distance of $d_{1}$m and $d_{2}$m from the sensor trajectory.

Based on the assumption that only one target of opportunity is tracked at any given time, the second target of opportunity is picked far away from the first target so that the second target will be in the sensor’s field of view for a reasonable time after completing the tracking of the first target. For instance, let us assume that the total number of time steps taken by the sensor for estimating the bias using one target of opportunity with the CV-CT model is *K*. Now, let us consider that the bias estimation is performed using two targets of opportunity. Denoting $K_{1}$ (where $K_{1}<K$) as the total number of time steps for which the first target of opportunity is used, we obtain the total number of time steps used for the second target of opportunity as $K_{2}=K-K_{1}$. Note that an equal number of time-steps are considered in both cases for a fair comparison. However, in practice, the total number of time steps depends on the sensor’s field of view and the target locations.

A better elevation bias estimate can be obtained while the target of opportunity is located closer to the sensor trajectory. To explain such a result, we analyze the reduction in PCRLB.

To validate the above notion, we provide the following experimental analysis. Denoting the first target of opportunity as t1 and the second target target of opportunity as t2, from Figure 3, we can write the x-coordinates as $x^{t1}=1000$ m and $x^{t2}=3000$ m. Note that, in presence of multiple targets of opportunity, we denote first, second, and third target of opportunity with lower-case letter ‘t1’, ‘t2’, and ‘t3’, respectively. Such notation is used to avoid conflict with the sampling time of sensors, which is denoted by the upper-case letter ‘*T*’. The y-coordinates are changed (while keeping $x^{t1}$ and $x^{t2}$ unchanged) to locate both the ‘t1’ and ‘t2’ at various relative distances from the sensor trajectory. A comparison of the PCRLB of $\gamma_{b}$ (in degree) at the end of the bias estimation is shown in Table 1. In this analysis, the performance of the CV model with two targets is compared with a CV-CT model with one target (t1), as described in the previous section.

From Table 1, the following conclusions are drawn:

- The addition of the second target of opportunity with different terrain height (i.e., different error in the assumed height) provides additional information to the estimator. Therefore, in most of the above simulation scenarios, estimation of ${\hat{\gamma }}_{b}$ with the CV model for sensor trajectory and two targets of opportunity provides better performance than that of the CT model for sensor trajectory and one target of opportunity;
- Bias estimation accuracy diminishes with the distance of the targets from the sensor. If the second target of opportunity is further away from the sensor trajectory, the additional information contributed to the bias estimation is insignificant. In such a scenario, the location of the first target of opportunity plays a significant role in the performance of bias estimation. As shown on the {8 and 9}-th row of Table 1, when the targets are relatively far away from the sensor trajectory, sensor trajectory with CV-CT model and one target of opportunity outperforms bias estimation obtained by the CV model along with two targets of opportunity;
- The bias estimation also depends on the error of the assumed height of the targets of opportunity. For our analysis in Table 1, different height errors are used for different targets. As shown on the 3-rd and 7-th rows of Table 1, we obtain different PCRLB estimates even with same *y* values (($y^{t1}=1300$, $y^{t2}=1900$) and ($y^{t1}=1900$, $y^{t2}=1300$)).

Additional results involving the Root Mean Square Error (RMSE) plots along with the scenarios of positioning the second target of opportunity on two opposing sides of the sensor trajectory are shown in Section 7.3.

From this analysis, we can conclude that we can obtain a better estimate of the biases by using multiple targets of opportunity without wasting additional time that we discussed in the previous section for the CV-CT model with a single target.

Although the bias estimation discussed in this section only considers targets of opportunity having known locations, we may need to pick an unknown stationary object as a target of opportunity. In the next section, bias estimation with an unknown location of the target of opportunity is introduced.

### 5.2. Unknown Location with Terrain Uncertainty

Following the same notations from Section 5.1, we consider a stationary target of opportunity with unknown $x^{to}$ and $y^{to}$ to estimate the bias. Let us denote the unknown stationary target and bias state as $x_{k}^{to}={\left[x_{k}^{to},y_{k}^{to},z_{k}^{to}\right]}^{T}$ and $b_{k}={\left[\theta_{b_{k}},\gamma_{b_{k}}\right]}^{T}$, respectively. In order to estimate both ${\hat{x}}^{to}$ and ${\hat{b}}_{k}$ simultaneously, we form the augmented state vector as $x_{k}^{\mathrm{aug}2}={\left[x_{k}^{to},y_{k}^{to},z_{k}^{to},\theta_{b_{k}},\gamma_{b_{k}}\right]}^{T}$. Our goal here is to estimate the augmented state vector ${\hat{x}}_{k}^{\mathrm{aug}2}={\left[{\hat{x}}_{k}^{to},{\hat{y}}_{k}^{to},{\hat{z}}_{k}^{to},{\hat{\theta }}_{b_{k}},{\hat{\gamma }}_{b_{k}}\right]}^{T}$, even though we are not interested in the target location.

We can write the initial augmented state vector $x_{1}^{\mathrm{aug}2}={\left[x_{1}^{to},y_{1}^{to},z_{m}^{to},0,0\right]}^{T}$, where $x_{1}^{to}$ and $y_{1}^{to}$ are converted position coordinates and $z_{m}^{to}$ is the assumed target height (equations are provided later in (29) and ((30) of Section 6.2). The measurement covariance matrix $R_{z}^{\mathrm{aug}2}=\mathrm{diag}\left(\sigma_{\theta }^{2},\sigma_{\gamma }^{2},\sigma_{z^{to}}^{2}\right)$ is used to find the initial covariance. Details about the construction of the Hessian matrix, i.e., $H_{z}^{\mathrm{aug}2}$, is shown in (A6). Once $H_{z}^{\mathrm{aug}2}$ and $R_{z}^{\mathrm{aug}2}$ are obtained, we obtain $J_{1}^{\mathrm{aug}2}$ from (22). Initial covariance is evaluated as $P_{1}^{\mathrm{aug}2}={\left(J_{1}^{\mathrm{aug}2}\right)}^{-1}$. Non-linear filter is used to update the state ${\hat{x}}_{k}^{\mathrm{aug}2}$.

In order to evaluate the bias estimation performance, PCRLB is evaluated following the formulation of Section 4. First, we evaluate the Hessian matrix $H_{k}^{\mathrm{aug}2}$ (details about the matrix construction is shown in (A5)) and the measurement covariance matrix $R_{k}^{\mathrm{aug}2}=\mathrm{diag}\left(\sigma_{\theta }^{2},\sigma_{\gamma }^{2}\right)$. With $H_{k}^{\mathrm{aug}2}$ and $R_{k}^{\mathrm{aug}2}$, the measurement contribution of the PCRLB is evaluated as $J_{z}^{\mathrm{aug}2}\left(k\right)=\left(H_{k}^{\mathrm{aug}2}\right){\left(R_{k}^{\mathrm{aug}2}\right)}^{-1}H_{k}^{\mathrm{aug}2}$. Substituting $H_{k}^{\mathrm{aug}2}$, $R_{k}^{\mathrm{aug}2}$ and $J_{z}^{\mathrm{aug}2}\left(k\right)$ into (9), PCRLB is evaluated.

In this section also, we study the possibility of improving bias estimation by,

- Changing sensor trajectory from the CV model to the CV-CT model;
- Choosing more than one target of opportunity in the sensor’s field of view.

#### 5.2.1. Change in Sensor Trajectory

For a known location of the target of opportunity, in Section 5.1.1, we concluded that a sensor trajectory comprised of the CV-CT model provides a relatively improved bias estimation when compared to that of the CV model. Now, considering the targets of opportunity with unknown locations are considered, we use PCRLB to explain the performance of bias estimation. Considering $H_{k}^{\mathrm{aug}2}$ from (A5) (Appendix A), the differentiation involving $\theta_{k}$ is dependent on $x_{k}^{rto}$ and $y_{k}^{rto}$. As a result, a sensor trajectory formed by the CV-CT model provides a reduced estimation error of ${\hat{\theta }}_{k}$ by reducing the relative distance between the sensor and the target of opportunity. Note that this was not the case with the known target location. However, for the differentiation involving $\gamma_{k}$, the relative height $z_{k}^{rto}$ is present in the numerator. As the terrain uncertainty is considered, a similar conclusion on a preferred trajectory can not be drawn for the estimation of ${\hat{\gamma }}_{k}$, as opposed to the estimation of ${\hat{\theta }}_{k}$.

Similar to Section 5.1, we now expand our analysis by introducing multiple targets of opportunity with unknown locations and terrain uncertainty.

#### 5.2.2. Bias Estimation with Multiple Targets of Opportunity

In this section, we investigate the possibility of improving the bias estimation by introducing multiple targets of opportunity in the sensor’s field of view. The sensor follows a trajectory formed by the CV model as opposed to the CV-CT model.

As discussed in Section 5.1, the bias estimation depends on the proximity of the targets of opportunity to the sensor trajectory. To analyze similar dependency for bias estimation with unknown locations, we perform a comparison of PCRLB. Let us consider the ground truth of x-coordinates of the first and second target of opportunity as $x^{t1}=1000$ m and $x^{t2}=3000$ m. For 3 different values of $y^{t1}$ and $y^{t2}$, we obtain three different locations of the target of opportunity based on its proximity to the sensor trajectory. Note that by location, we refer to the ground truth needed to evaluate the PCRLB.

Let us analyze the PCRLB evaluations from Table 2. Firstly, we can draw the following conclusions for ${\hat{\theta }}_{b_{k}}$:

- When the sensor follows a trajectory formed by the CV-CT model, one target of opportunity is sufficient to estimate the bearing bias. See Section 5.1.2 for explanations.

Secondly, the following conclusions can be drawn for ${\hat{\gamma }}_{b_{k}}$:

- When both the targets of opportunity are relatively far away from the sensor trajectory, a better estimation of ${\hat{\gamma }}_{b_{k}}$ is obtained when the sensor follows a trajectory formed by CV-CT model. The reason behind such result can be attributed to the reduction in relative distance $x_{k}^{rto}$ and $y_{k}^{rto}$, which, in turn, reduces the PCRLB;
- When t1 is away from the sensor trajectory, we draw the same conclusions as the known locations of the targets of opportunity, discussed in Section 5.1.

Following the above discussions, following the CV-CT model with one target of opportunity is a better choice than following the CV model with two targets of opportunity. However, adding one more target of opportunity after completing the CV-CT model with the first target of opportunity on the way to the destination with the CV model will help to improve the elevation bias estimate.

## 6. Tracking Target with Terrain Uncertainty and Sensor Biases

In this section, we provide the algorithm for tracking stationary and moving targets with terrain uncertainty and sensor biases. Note that even if we perform the bias estimation using targets of opportunity, we do not find the exact bias to do the de-biasing before passing the measurements to the tracker. The variance of the bias at the start of the tracking will be larger if we do not perform the step proposed in Section 5. However, the tracking of the target of interest should incorporate possible biases.

Since false alarms are not considered in this paper, the tracking includes initialization and filtering.

### 6.1. Bias Compensation

For bias compensation, the bias state and the standard deviations are required to be known. In this work, we consider two following cases: bias compensation based on a bias prior and bias estimation using a target of opportunity.

- Let us assume that the bias state is known with a reasonable level of accuracy. In this text, the term reasonable level of accuracy does not refer to any formal definition of accuracy. We denote such a state with the superscript ‘deduced’. In other words $b^{\mathrm{deduced}}=\left[\theta_{b}^{\mathrm{deduced}},\gamma_{b}^{\mathrm{deduced}}\right]$ is known a-priori. Additionally, the corresponding bias standard deviations $\sigma_{\theta_{b}}^{\mathrm{deduced}}$ and $\sigma_{\gamma_{b}}^{\mathrm{deduced}}$ are known;
- However, in most applications, obtaining such prior information about the bias state and the standard deviation is not practical. Hence, estimating the bias state $\hat{b}=\left[{\hat{\theta }}_{b},{\hat{\gamma }}_{b}\right]$ and ${\hat{\sigma }}_{\theta_{b}}$, ${\hat{\sigma }}_{\gamma_{b}}$ using a target of opportunity is a more suitable alternative, which is discussed detail in Section 5.

Once the bias state and the corresponding standard deviations are obtained, by either of the two ways described above, we obtain the bias compensated measurements $z_{k}^{c}$ as,

(27) $$\begin{array}{c} z_{k}^{c}=z_{k}-z_{k}^{\mathrm{prior}} \end{array}$$

where $z_{k}^{\mathrm{prior}}$ is the measurement obtained by substituting $b_{k}$ with $b_{k}^{\mathrm{deduced}}$ or ${\hat{b}}_{k}$ in (5). Similarly, the modified measurement covariance matrix $R_{k}^{c}$ is obtained as,

(28) $$\begin{array}{ccc} R_{k}^{c} & = & \left[\begin{array}{cc} \sigma_{\theta }^{2}+{\hat{\sigma }}_{\theta_{b}}^{2} & 0\\ 0 & \sigma_{\gamma }^{2}+{\hat{\sigma }}_{\gamma_{b}}^{2} \end{array}\right] \end{array}$$

When the bias standard deviations are known a priori, ${\hat{\sigma }}_{\theta_{b}}$ and ${\hat{\sigma }}_{\gamma_{b}}$ are substituted with $\sigma_{\theta_{b}}^{\mathrm{deduced}}$ and $\sigma_{\gamma_{b}}^{\mathrm{deduced}}$, respectively.

### 6.2. Initialization

Recalling the discussions from Section 3, the height of the ground target is obtained from DTED and it can be used for track initialization. Note that we should not use this information again in the filtering steps to avoid double counting. From the target-sensor geometry shown in Figure 1, we can write the converted Cartesian coordinates of the target state as,

(29) $$\begin{array}{ccc} x_{1}^{t} & = & \frac{z_{m}^{t}}{\tan(\gamma_{1})}\sin\left(\theta_{1}\right) \end{array}$$

(30) $$\begin{array}{ccc} y_{1}^{t} & = & \frac{z_{m}^{t}}{\tan(\gamma_{1})}\cos\left(\theta_{1}\right) \end{array}$$

With the *x* and *y* coordinates from (29) and (30) along with $z_{m}^{t}$, the initial state of the ground target is written as $x_{1}^{t}={\left[x_{1}^{t},0,y_{1}^{t},0,z_{m}^{t}\right]}^{T}$. Note that the initial velocity along the *x* and *y*-axis, ${\dot{x}}_{1}^{t}$ and ${\dot{y}}_{1}^{t}$, are considered to be 0 in this one-point initialization [33]. Similarly, for the stationary target we can write $x_{1}^{t}={\left[x_{1}^{t},y_{1}^{t},z_{m}^{t}\right]}^{T}$.

In order to initialize the covariance $P_{1}$, let us first introduce the covariance associated with measurements, including the terrain height, as $R_{z}^{c}=\mathrm{diag}\left(\sigma_{\theta }^{2}+{\hat{\sigma }}_{\theta_{b}}^{2},\sigma_{\gamma }^{2}+{\hat{\sigma }}_{\gamma_{b}}^{2},\sigma_{z^{t}}^{2}\right)$. Similar to the other sections of this paper, we reserve the use of subscript ‘*z*’ to denote the initial time step when the height information is considered to evaluate the measurement covariance and the Hessian matrices. Note that $R_{z}^{c}$ is different from that of the noise covariance $R_{k}^{c}$, as the terrain uncertainty is considered only for the filter initialization. Now following (9), we evaluate $J_{1}$ to obtain $P_{1}=J_{1}^{-1}$. First, the measurement contribution for initialization $J_{z}\left(1\right)$ can be evaluated as $J_{z}\left(1\right)=H_{z}^{T}{\left(R_{z}^{c}\right)}^{-1}H_{z}$, where the initial Jacobian matrix $H_{z}$ is,

(31) $$\begin{array}{ccc} H_{z} & = & \left[\begin{array}{ccccc} \frac{\partial \theta_{1}}{\partial x_{1}^{t}} & \frac{\partial \theta_{1}}{\partial {\dot{x}}_{1}^{t}} & \frac{\partial \theta_{1}}{\partial y_{1}^{t}} & \frac{\partial \theta_{1}}{\partial {\dot{y}}_{1}^{t}} & \frac{\partial \theta_{1}}{\partial z_{1}^{t}}\\ \frac{\partial \gamma_{1}}{\partial x_{1}^{t}} & \frac{\partial \gamma_{1}}{\partial {\dot{x}}_{1}^{t}} & \frac{\partial \gamma_{1}}{\partial y_{1}^{t}} & \frac{\partial \gamma_{1}}{\partial {\dot{y}}_{1}^{t}} & \frac{\partial \gamma_{1}}{\partial z_{1}^{t}}\\ \frac{\partial z_{1}^{t}}{\partial x_{1}^{t}} & \frac{\partial z_{1}^{t}}{\partial {\dot{x}}_{1}^{t}} & \frac{\partial z_{1}^{t}}{\partial y_{1}^{t}} & \frac{\partial z_{1}^{t}}{\partial {\dot{y}}_{1}} & \frac{\partial z_{1}^{t}}{\partial z_{1}^{t}} \end{array}\right] \end{array}$$

Evaluating the partial derivatives from (31), we can write the analytical form of $H_{z}$ as,

(32) $$\begin{array}{ccc} H_{z} & = & \left[\begin{array}{ccccc} \frac{y_{1}^{r}}{x_{1}^{r^{2}}+y_{1}^{r^{2}}} & 0 & \frac{-x_{1}^{r}}{x_{1}^{r^{2}}+y_{1}^{r^{2}}} & 0 & 0\\ \frac{-x_{1}^{r}z_{1}^{r}}{\sqrt{x_{1}^{r^{2}}+y_{1}^{r^{2}}}\left(x_{1}^{r^{2}}+y_{1}^{r^{2}}+z_{1}^{r^{2}}\right)} & 0 & \frac{-y_{1}^{r}z_{1}^{t}}{\sqrt{x_{1}^{r^{2}}+y_{1}^{r^{2}}}\left(x_{1}^{r^{2}}+y_{1}^{r^{2}}+z_{1}^{r^{2}}\right)} & 0 & \frac{\sqrt{x_{1}^{r^{2}}+y_{1}^{r^{2}}}}{x_{1}^{r^{2}}+y_{1}^{r^{2}}+z_{1}^{r^{2}}}\\ 0 & 0 & 0 & 0 & 1 \end{array}\right] \end{array}$$

where $x_{1}^{r}$, $y_{1}^{r}$ and $z_{1}^{r}$ denote the coordinates of the relative state at time step k=1 ($x_{1}^{rt}=x_{1}^{t}-x_{1}^{o}$).

For the moving target, maximum possible target velocity, $v_{max}$, is used to evaluate $J_{p}=\mathrm{diag}\left(0,\frac{1}{v_{max}^{2}},0,\frac{1}{v_{max}^{2}},0\right)$. The initial covariance $P_{1}$ is obtained by $P_{1}={\left(J_{z}\left(1\right)+J_{p}\right)}^{-1}$. Evidently, for the stationary target $J_{1}=J_{z}\left(1\right)$ and initial covariance $P_{1}=J_{z}^{-1}$. Note that the Hessian matrix, $H_{z}^{S}$, for the stationary target is expressed as,

(33) $$\begin{array}{ccc} H_{z}^{S} & = & \left[\begin{array}{ccc} \frac{y_{1}^{r}}{x_{1}^{r^{2}}+y_{1}^{r^{2}}} & \frac{-x_{1}^{r}}{x_{1}^{r^{2}}+y_{1}^{r^{2}}} & 0\\ \frac{-x_{1}^{r}z_{1}^{r}}{\sqrt{x_{1}^{r^{2}}+y_{1}^{r^{2}}}\left(x_{1}^{r^{2}}+y_{1}^{r^{2}}+z_{1}^{r^{2}}\right)} & \frac{-y_{1}^{r}z_{1}^{r}}{\sqrt{x_{1}^{r^{2}}+y_{1}^{r^{2}}}\left(x_{1}^{r^{2}}+y_{1}^{r^{2}}+z_{1}^{r^{2}}\right)} & \frac{\sqrt{x_{1}^{r^{2}}+y_{1}^{r^{2}}}}{x_{1}^{r^{2}}+y_{1}^{r^{2}}+z_{1}^{r^{2}}}\\ 0 & 0 & 1 \end{array}\right] \end{array}$$

The superscript ‘*S*’ in $H_{z}^{S}$, of (33), indicates the stationary target.

### 6.3. Filtering

Once the filter is initialized and the measurements are bias compensated, UKF is used to handle the non-linearity and obtain the estimated target state ${\hat{x}}_{k}^{t}$ and the associated covariance ${\hat{P}}_{k}$ [30]. The effect of terrain uncertainty is already considered in obtaining the initial state and the associated covariance. Hence, only the bearing and elevation measurements are used to update the state. Non-linear filtering uses (1) for the state prediction and (5) for the measurement update. Proposed filtering approach using UKF involves the following steps: calculation of the sigma points, measurement prediction and update. These processes are well known and not discussed in this paper.

Now, in the next section, we present the simulation results for tracking both the stationary and moving targets with terrain uncertainty using a biased angle-only sensor.

## 7. Simulations

### 7.1. Parameters

**Figure 4:**
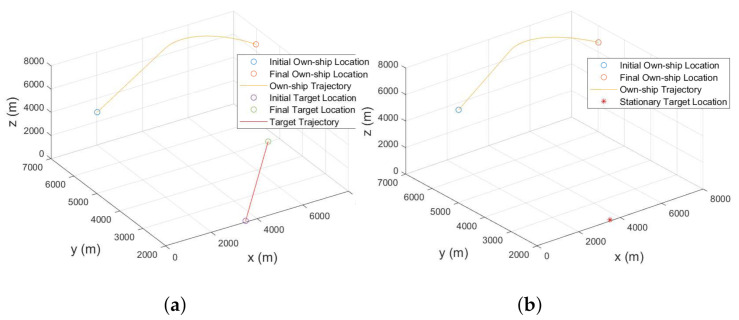
Geometry of the airborne sensor and the ground target with terrain uncertainty. (**a**) Target moves in nearly constant velocity. (**b**) Target remains stationary.

The target-sensor geometry is shown in Figure 4. We consider both mobile and stationary targets for this simulation as shown in Figure 4a,b, respectively. The own-ship follows a trajectory formed by the CV-CT model. Parameters used in this simulation are provided in Table 3. Note that in all initial states, the positions along *x*, *y*, and *z*-coordinates are in meter whereas the velocities $\dot{x}$, $\dot{y}$ and $\dot{z}$ are in m/s.

Considering $\sigma_{z^{t}}=10$ m, we obtain the error associated with the terrain uncertainty as $v^{z}∼N\left(v^{z};0,10^{2}\right)$. The moving target trajectory follows the CV model.

### 7.2. Performance Bound

**Figure 5:**
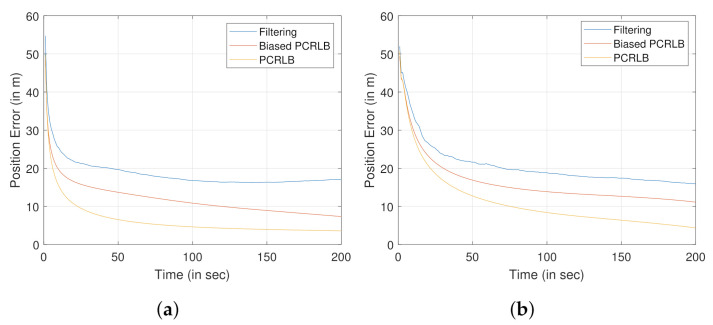
Comparison of the performance bounds. (**a**) Stationary ground target. (**b**) Mobile ground target.

Figure 5a,b show the position error plots for tracking the stationary and moving ground target, respectively. The position errors are obtained by performing square root on the sum of the diagonal elements of the covariance matrix (elements representing the x, y, and z coordinates). Position errors for both the biased PCRLB (see Section 4.4) and the PCRLB (see Section 4.2) are evaluated to compare with that of the proposed filtering approach. In order to evaluate the position error obtained by the estimated target states using the proposed filtering approach, we performed 500 Monte Carlo simulations.

From Figure 5, it is clear that the biased PCRLB would provide a tighter bound on position error when compared to the standard PCRLB.

### 7.3. Bias Estimation

In this section, we analyze the bias estimation for the true values of $\theta_{b_{k}}=1^{\circ }$ and $\gamma_{b_{k}}=1^{\circ }$. Non-linear filtering based on UKF is used to estimate the bias state using the measurements obtained from the stationary target of opportunity. Note that, all the targets of opportunity considered in this section have terrain uncertainty. We show the simulation results for the two following scenarios. In scenario 1, we consider the location of the target of opportunity is known with terrain uncertainty. In scenario 2, the location of the target of opportunity is unknown.

#### 7.3.1. Scenario 1

**Figure 6:**
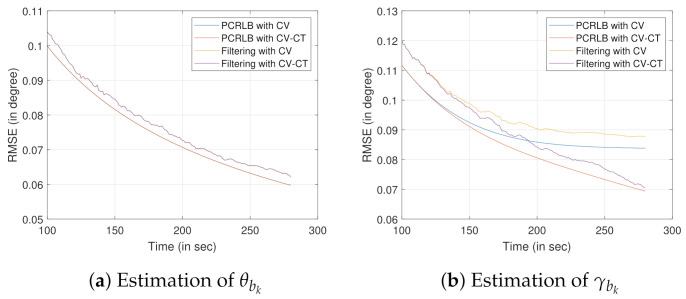
RMSE evaluation for bias estimation using one target of opportunity with known location and terrain uncertainty.

Recalling the discussions from Section 5, one (or multiple) target of opportunity is chosen to estimate the bias. Figure 6 shows the RMSE values for the bias estimation using one stationary target of opportunity with known location. We consider the initial state of the target of opportunity as $x_{1}^{to}={\left[x_{1}^{to},y_{1}^{to},z_{1}^{to}\right]}^{T}={\left[1000,1200,100\right]}^{T}$. The initial state of the own-ship is $x_{1}^{o}={\left[x_{1}^{o},{\dot{x}}_{1}^{o},y_{1}^{o},{\dot{y}}_{1}^{o},z_{1}^{o}\right]}^{T}={\left[0,10,1000,0,2500\right]}^{T}$.

To simulate the effect of change in sensor trajectory, discussed in Section 5.1.1, the CV model and the CV-CT model are used in this simulation. We obtain $K_{CV}=\frac{1000}{10}=100$ sec as the time-step where the own-ship trajectory switches from the CV model to the CT model (when the CV-CT model is considered). As discussed in Section 5.1.1, the CV-CT model reduces the relative distances between the sensor and the target of opportunity. This leads to the reduction in $x_{k}^{rto}$ and $y_{k}^{rto}$, causing the reduction in PCRLB. Hence, justifiably, the CV-CT model improves the estimate ${\hat{\gamma }}_{b_{k}}$ when compared to that of the sensor trajectory formed by the CV model. Recalling the discussions from Section 5.1.1, differentiation with respect to $\theta_{k}$ for obtaining the Hessian matrix is constant. As a result, for estimating ${\hat{\theta }}_{b_{k}}$ there is no change in the RMSE evaluation (or PCRLB) while the trajectories are changed. Note that the difference in RMSE, for both the CV and the CV-CT, is negligible before the time step k=100 s. Hence, to produce a meaningful RMSE comparison for the said sensor trajectories, the last 181 time steps (starting from k=100 s) are shown in Figure 6.

**Figure 7:**
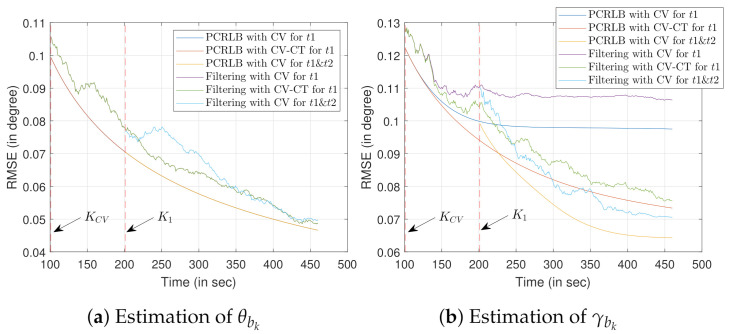
RMSE evaluation for bias estimation using two targets of opportunity with a known location. Terrain uncertainty is present with the target height information.

Now, we simulate the bias estimation results considering multiple targets of opportunity. Recalling the conclusions from Section 5.1.2, two targets of opportunity with known x and y coordinates, each having terrain uncertainty, are chosen. The sensor follows a trajectory formed by the CV model. The first target of opportunity is initialized as $x_{1}^{t1}={\left[x_{1}^{t1},y_{1}^{t1},z_{1}^{t1}\right]}^{T}={\left[1000,1600,100\right]}^{T}$. Keeping $z_{1}^{t2}=z_{1}^{t1}$, the second target of opportunity is initialized as $x_{1}^{t2}={\left[x_{1}^{t2},y_{1}^{t2},z_{1}^{t2}\right]}^{T}={\left[3000,1200,100\right]}^{T}$. For this simulation, we assume that t2 is introduced in the sensor’s field of view at $K_{1}=2\times K_{CV}$. Evidently, from Figure 7b, it is clear that the RMSE, as well as the PCRLB, for the estimate ${\hat{\gamma }}_{b_{k}}$ drops when t2 is introduced at $K_{1}$ time-step. However, the addition of t2 has no impact on the RMSE of ${\hat{\theta }}_{b_{k}}$ estimate.

The RMSE (as well as the PCRLB) obtained using one target of opportunity, while the sensor follows the CV-CT model, are also plotted. When compared with the PCRLB obtained using two targets of opportunity with the CV model, one target of opportunity with the CV-CT model provides inferior ${\hat{\gamma }}_{b_{k}}$ estimates. The reason behind such simulation results can be largely attributed to the information gain facilitated by the addition of the target of opportunity $t_{2}$. However, for the $\theta_{b_{k}}$ estimation no performance difference is observed. This result further confirms the conclusions obtained from Figure 6a, where we verified the change in the sensor trajectory does not impact on the $\theta_{b_{k}}$ estimation. Figure 7 also validates the conclusions drawn in Section 5.1.2. 100 Monte Carlo runs are performed to obtain these simulation results. Note that the differences in RMSE and PCRLB, for all the considered cases, are negligible before the time step k=100 s. Hence, to produce a meaningful comparison between the bias estimation approaches, only the last 360 time steps (starting from k=100 s) are considered in Figure 7.

Next, we analyze the significance of its close proximity to the sensor trajectory. We consider the second target of opportunity is introduced at $K_{1}=200$ s time step.

**Figure 8:**
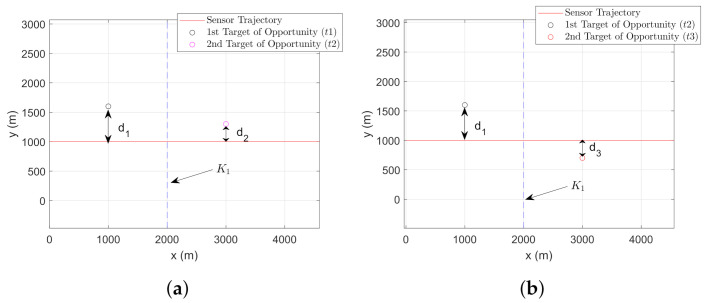
Sensor trajectory formed by the CV model to estimate bias using two known targets of opportunity with additional terrain uncertainty. (**a**) Second target of opportunity (t2) located $d_{2}$m away from sensor trajectory. (**b**) Second target of opportunity (t3) located $d_{3}$m away the sensor trajectory.

Table 1 (from Section 5.1.2) showed the RMSE comparison while the t2 is located either close or away from the sensor trajectory. Now we consider the possibility of t2 to be located on either side of the sensor trajectory. In Figure 8a, t2 is located at a distance of $d_{2}=300$ m from the sensor trajectory. The initial state vector of t2, is given as $x_{1}^{t2}={\left[x_{1}^{t2},y_{1}^{t2},z_{1}^{t2}\right]}^{T}={\left[3000,1300,100\right]}^{T}$. Whereas, we consider t3 as the second target of opportunity being located $d_{3}=300$ m away from the sensor trajectory, with the initial state vector expressed as $x_{1}^{t3}={\left[x_{1}^{t3},y_{1}^{t3},z_{1}^{t3}\right]}^{T}={\left[3000,700,100\right]}^{T}$. We consider $\sigma_{z^{t2}}=\sigma_{z^{t3}}=10$ m, for the simulation. Once $x_{1}^{t2}$ and $x_{1}^{t3}$ are initialized, the proposed filtering approach is used to estimate the bias state ${\hat{b}}_{k}$. Note that the first target of opportunity $\left(t1\right)$ is located at a distance of $d_{1}=600$ m from the sensor trajectory.

**Figure 9:**
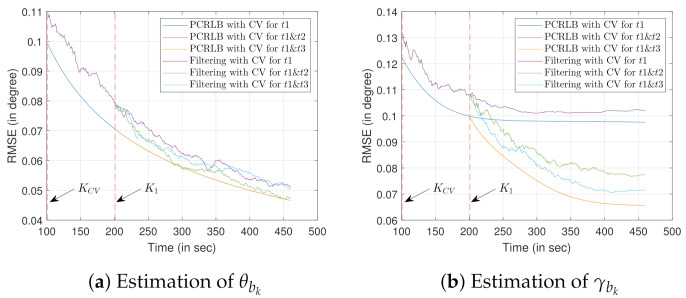
RMSE evaluation for bias estimation using two targets of opportunity with known location and terrain uncertainty. The second target of opportunity is located on either side of the sensor trajectory.

The PCRLB is a function of the relative distance between the sensor and the target of opportunity. Hence, the bias estimates are not impacted by which side the target of opportunity is located with respect to the sensor trajectory. RMSE evaluation results, presented in Figure 9b, validate such notion. Close proximity of the target of opportunity to the sensor trajectory reduces the relative distances, which, in turn, impacts the bias estimation as shown in Table 1.

Similar to Figure 7, to produce a meaningful comparison between the bias estimation approaches, only the last 360 time steps (starting from k=100 s) are considered in Figure 9. In total, 100 Monte Carlo simulations are performed to obtain all the Figures shown in Scenario 1.

#### 7.3.2. Scenario 2

**Figure 10:**
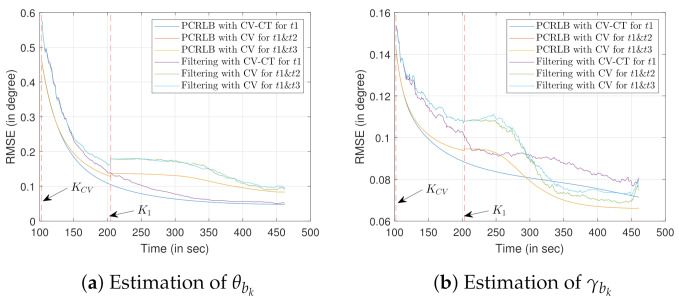
RMSE evaluation for bias estimation using two targets of opportunity with unknown location and terrain uncertainty. The second target of opportunity is located on either side of the sensor trajectory.

We consider two targets of opportunity along with two types of sensor trajectories that are formed with the CV and the CV-CT models. Figure 10 shows the RMSE and PCRLB values of the bias estimation when the x and y coordinates of the targets of opportunity are unknown.

Ground truth for the first target of opportunity (t1) is $x_{1}^{t1}={\left[x_{1}^{t1},y_{1}^{t1},z_{1}^{t1}\right]}^{T}=[1000,1400,$ ${100]}^{T}$. The ground truth for the second target of opportunity, which is $d_{2}=300$ m away from the sensor trajectory, $x_{1}^{t2}={\left[x_{1}^{t2},y_{1}^{t2},z_{1}^{t2}\right]}^{T}={\left[3000,1400,100\right]}^{T}$. Similarly, the ground truth of the target of opportunity t3, located $d_{3}=300$ m away from the sensor trajectory on the opposite side, can be expressed as $x_{1}^{t3}={\left[x_{1}^{t3},y_{1}^{t3},z_{1}^{t3}\right]}^{T}={\left[3000,600,100\right]}^{T}$. Note that only one of the two targets from t2 and t3 is used with target t1. The proposed filter is initialized with the converted measurements obtained at time step k=1.

Similar to Scenario 1, the own-ship trajectory switches from the CV to the CT model at the time step $K_{CV}=100$ s (shown in Figure 10a). The sensor starts tracking the second target of opportunity at $K_{1}=200$ s time step. Similar to Scenario 1, it is assumed that the second target of opportunity (either t2 or t3) is made available to the sensor’s field of view at $K_{1}=2\times K_{CV}$ time step. Unlike the estimation of $\theta_{b_{k}}$ in scenario 1, RMSE (as well as the PCRLB) obtained by using one target of opportunity while following the CV-CT model is lower than that of using two targets of opportunity and the CV model. As the x and y coordinates are unknown for both the targets of opportunity, adding t2 (or t3) in addition to that of t1 does not improve the estimation of $\theta_{b_{k}}$. However, as the height information of both the targets of opportunity is known, the addition of t2 (or t3) improves the estimation of $\gamma_{b_{k}}$, as shown in Figure 10b. However, because of the presence of the terrain uncertainty for the first few time steps, the CV-CT model with one target of opportunity provides a superior estimation of $\gamma_{b_{k}}$. With time, as the filter converges, the improvement caused by the addition of the second target of opportunity while following the CV model becomes evident. Similar to that of Section 1, no improvement is observed in the PCRLB estimates while using t2 as opposed to t3.

Similar to Scenario 1, only the last 360 time steps (starting from k=100 s) are considered in Figure 10 to produce a meaningful comparison between the bias estimation approaches. We performed 100 Monte Carlo simulations to obtain all the results of Scenario 2.

Note that the ground truth of the bearing and the elevation biases of $\theta_{b_{k}}=1^{\circ }$ and $\gamma_{b_{k}}=1^{\circ }$ are chosen to validate our proposed bias estimation approaches. This is not to be confused with the ground truth values of $\theta_{b_{k}}=3^{\circ }$ and $\gamma_{b_{k}}=1^{\circ }$, as introduced in Table 3, for tracking the original target. In the next section, for bias compensation, we choose the bias estimation approach involving two targets of opportunities with known x and y coordinates and the CV sensor trajectory.

### 7.4. Tracking Using Angle-Only Measurements

Let us recall the original tracking problem, where a ground target with terrain uncertainty is tracked using a biased airborne angle-only sensor. Parameters for both the sensor and the target initialization are presented in Table 3. The estimates of the biases from a sample run are ${\hat{\theta }}_{b_{k}}=2.89^{\circ }$ and ${\hat{\gamma }}_{b_{k}}=1.03^{\circ }$ with the associated variances $\sigma_{{\hat{\theta }}_{b_{k}}}=0.0597^{\circ }$ and $\sigma_{{\hat{\gamma }}_{b_{k}}}=0.0834^{\circ }$.

**Figure 11:**
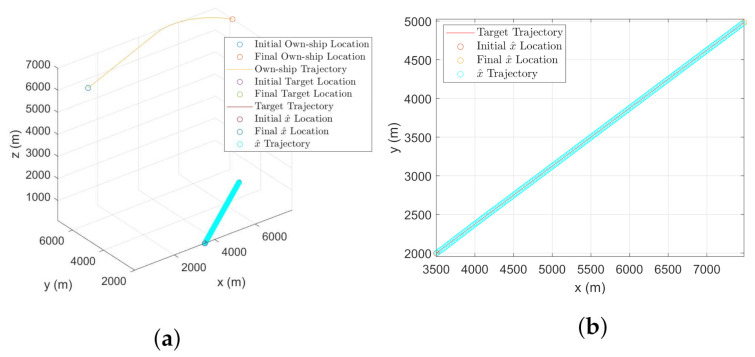
Tracking a mobile ground target with terrain uncertainty using a biased angle-only airborne sensor. (**a**) 3D CCS representation of the target-sensor geometry with the estimated trajectory. (**b**) 2D CCS representation of the target and the estimated trajectory.

**Figure 12:**
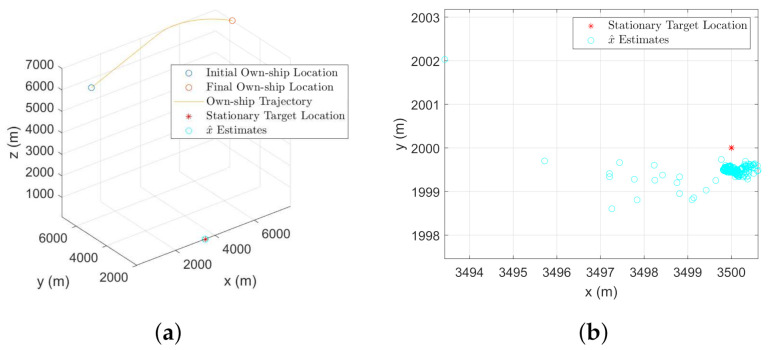
Tracking a stationary ground target with terrain uncertainty using an airborne angle-only sensor. (**a**) 3D CCS representation of the target own-ship geometry along with the estimates. (**b**) 2D CCS representation of the stationary target and the estimates.

**Figure 13:**
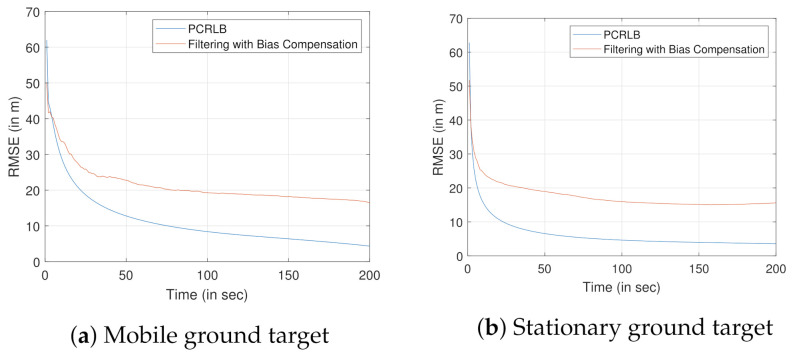
RMSE evaluation for tracking a ground target using airborne sensor platform with angle-only bias-compensated measurements.

**Figure 14:**
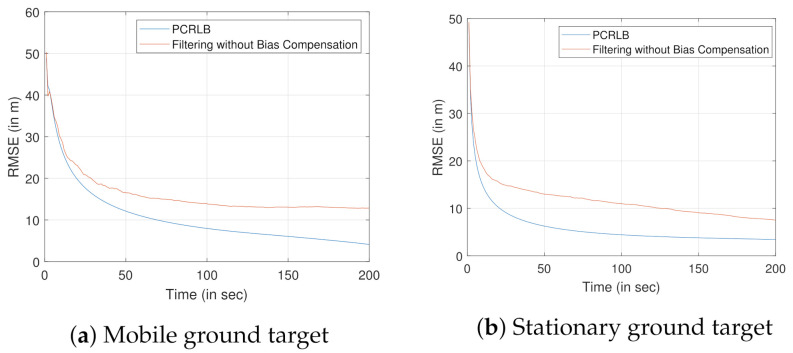
RMSE evaluation for tracking a ground target using an airborne sensor platform with unbiased angle-only measurements.

Once bias compensation is performed to reduce the bias uncertainty, proposed filtering approach is used for tracking both the moving or stationary target with terrain uncertainty. Figure 11a and Figure 12a show the 3D CCS representation of the sensor trajectories and the estimated trajectories (or the estimates for the stationary target). The corresponding 2D CCS representations are shown in Figure 11b and Figure 12b, respectively. The RMSE plots along with the corresponding PCRLBs are shown in Figure 13. To establish a baseline, we evaluate the RMSE and the PCRLB considering no measurement bias, i.e., $\theta_{b_{k}}=0^{\circ }$ and $\gamma_{b_{k}}=0^{\circ }$. Corresponding RMSE plots considering both moving and stationary ground targets, with terrain uncertainty, are shown in Figure 14a,b, respectively.

The effect of bias uncertainty on the estimates is observed in Figure 12b. A handful of initial estimates are away from the original target. However, as the filter converges, the effect of bias uncertainty is minimized. Although a similar estimation error is caused by the bias uncertainty while considering the moving target tracking, it is not clearly observed in Figure 11b because of the scaling of the y-axis. The RMSE analysis, shown in Figure 13, validates our approach for both moving and stationary targets. All the simulation results in this section are obtained by performing an average of over 500 Monte Carlo runs.

**Figure 15:**
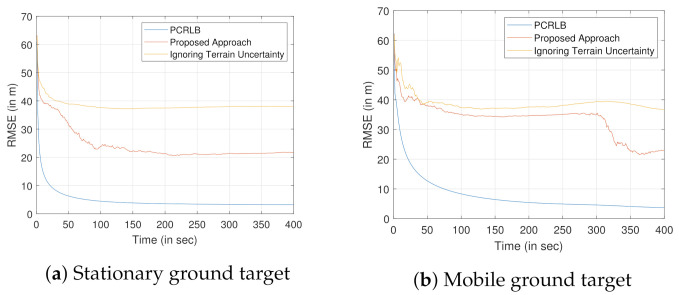
Comparison of the RMSE of the proposed approach with an approach that ignores the terrain uncertainty, when the $\sigma_{z^{t}}=30$ m.

Now, we analyze the performance of the proposed approach with a conventional method, where the terrain uncertainty is ignored, for tracking both the stationary and moving ground targets. The RMSE comparison with $\sigma_{z^{t}}=30$ m is shown in Figure 15. As expected, our proposed approach provides better RMSE for both the stationary and the moving ground targets. Although we ignore the terrain uncertainty, we use a wrong value for the height of the target. Hence, it results in a bias in the estimate and that bias will not even be reduced with more measurements. That is what we see in Figure 15 for both cases. For moving target, because of the target sensor geometry, significant change in the RMSE is noticed only after the first 300 s. Both the simulations were performed by taking an average of over 100 Monte Carlo runs.

## 8. Conclusions

In this paper, we have considered the 3-D tracking of a ground target with terrain uncertainty using a biased angle-only airborne sensor. We derived the PCRLB bound for the problem with sensor bias and terrain uncertainty. We provided a bias gradient-based PCRLB formulation to find a tighter bound under biases. We showed that the biased PCRLB provides a tighter lower bound when compared with the PCRLB while evaluating position error. Using the derived PCRLB, we proposed a method to pick an optimal target(s) of opportunity and optimal platform trajectory to estimate the bias. We demonstrated that tracking of a ground target in 3-D could be performed with biased angle-only measurements using UKF as a preferred non-linear filtering method.

## Figures and Tables

**Table 1:** PCRLB (in degrees) of $\gamma_{b}$ (in degrees) with different sensor trajectories and various locations of two targets of opportunity.

y-Coordinates		Sensor Trajectories	
$y^{t2}$	$y^{t1}$	CV Model	CV-CT Model
	1300	0.0621	0.0703
1300	1600	0.0656	0.0734
	1900	0.0687	0.0742
	1300	0.0659	0.0703
1600	1600	0.0701	0.0734
	1900	0.0740	0.0742
	1300	0.0691	0.0703
1900	1600	0.0740	0.0734
	1900	0.0786	0.0742

**Table 2:** PCRLB (in degrees) for $\theta_{b_{k}}$ and $\gamma_{b_{k}}$ estimation by changing sensor trajectories for various true locations of the 2 targets of opportunity.

		Sensor Trajectories			
y-Coordinates		for ${\hat{\theta }}_{b_{k}}$ (in Degrees)		for ${\hat{\gamma }}_{b_{k}}$ (in Degrees)	
$y^{t1}$	$y^{t2}$	CV Model	CV-CT Model	CV Model	CV-CT Model
	1800	0.1083	0.0480	0.0726	0.0714
1400	1600	0.0944	0.0480	0.0694	0.0714
	1400	0.0828	0.0480	0.0661	0.0714
	1800	0.1202	0.0476	0.0771	0.0733
1600	1600	0.1054	0.0476	0.0733	0.0733
	1400	0.0892	0.0477	0.0695	0.0733
	1800	0.1286	0.0472	0.0817	0.0740
1800	1600	0.1107	0.0472	0.0770	0.0739
	1400	0.0931	0.0473	0.0729	0.0740

**Table 3:** Parameters.

Parameters	Value
Initial state of the own-ship $\left(x_{1}^{o}=\left[x_{1}^{o},{\dot{x}}_{1}^{o},y_{1}^{o},{\dot{y}}_{1}^{o},z_{1}^{o}\right]\right)$	${\left[20,40,5000,15,7000\right]}^{T}$
Initial state of the mobile target $\left(x_{1}^{t}=\left[x_{1}^{t},{\dot{x}}_{1}^{t},y_{1}^{t},{\dot{y}}_{1}^{t},z_{1}^{t}\right]\right)$	${\left[3500,20,2000,15,100\right]}^{T}$
Initial state of the stationary target $\left(x_{1}^{t}=\left[x_{1}^{t},y_{1}^{t},z_{1}^{t}\right]\right)$	${\left[3500,2000,100\right]}^{T}$
Standard deviation of terrain uncertainty ($\sigma_{z^{t}}$)	10 m
Bearing measurement noise standard deviation ($\sigma_{\theta }$)	$0.4^{\circ }$
Elevation measurement noise standard deviation ($\sigma_{\gamma }$)	$0.2^{\circ }$
Sampling time (*T*)	1 s
Maximum velocity of the ground target ($v_{max}$)	35 m/s
Bearing bias ($\theta_{b_{k}}$)	$3^{\circ }$
Elevation bias ($\gamma_{b_{k}}$)	$1^{\circ }$
Total simulation time	200 s

## Data Availability

Not applicable.

## Appendix A

Let us denote the state transition matrices for the CV and CT motion as $F_{CV}$ and $F_{CT}$. We also denote the state of the ground target and the own-ship as $x_{k}^{t}={\left[x_{k}^{t},{\dot{x}}_{k}^{t},y_{k}^{t},{\dot{y}}_{k}^{t},z_{k}^{t}\right]}^{T}$ and $x_{k}^{o}={\left[x_{k}^{o},{\dot{x}}_{k}^{o},y_{k}^{o},{\dot{y}}_{k}^{o},z_{k}^{o}\right]}^{T}$. Given the sampling time T and turn rate ω, we can write,

(A1) $$F_{CV}=\left[\begin{array}{ccccc} 1 & T & 0 & 0 & 0\\ 0 & 1 & 0 & 0 & 0\\ 0 & 0 & 1 & T & 0\\ 0 & 0 & 0 & 1 & 0\\ 0 & 0 & 0 & 0 & 1 \end{array}\right]\;F_{CT}=\left[\begin{array}{ccccc} 1 & \frac{\sin(\omega T)}{\omega } & 0 & \frac{\cos(\omega T)-1}{\omega } & 0\\ 0 & \cos(\omega T) & 0 & -\sin(\omega T) & 0\\ 0 & \frac{1-\cos(\omega T)}{\omega } & 1 & \frac{\sin(\omega T)}{\omega } & 0\\ 0 & \sin(\omega T) & 0 & \cos(\omega T) & 0\\ 0 & 0 & 0 & 0 & 1 \end{array}\right]$$

Note, $F_{CV}$ is used to model the state transition of both the own-ship and the ground target (when the ground target moves with nearly CV). However, $F_{CT}$ is used to model the state transition of the own-ship for the bias estimation when the CV-CT model is used.

In order to model the system dynamics of the target, gain matrix *G* is required to obtain the process noise. Gain matrix G is written as,

(A2) $$\begin{array}{ccc} G & = & \left[\begin{array}{cc} \frac{T^{2}}{2} & 0\\ T & 0\\ 0 & \frac{T^{2}}{2}\\ 0 & T\\ 0 & 0 \end{array}\right] \end{array}$$

We denote the relative state vector as $x_{k}^{r}=x_{k}^{t}-x_{k}^{o}$, where $x_{k}^{t}$ and $x_{k}^{o}$ are the state vector of the target and the own-ship, respectively. Considering the angle-only measurements $z_{k}=\left[\theta_{k},\gamma_{k}\right]$, the Jacobian matrix for the measurements can be formed as,

(A3) $$\begin{array}{ccc} H_{k} & = & \left[\begin{array}{ccccc} \frac{\partial \theta_{k}}{\partial x_{k}^{t}} & \frac{\partial \theta_{k}}{\partial {\dot{x}}_{k}^{t}} & \frac{\partial \theta_{k}}{\partial y_{k}^{t}} & \frac{\partial \theta_{k}}{\partial {\dot{y}}_{k}^{t}} & \frac{\partial \theta_{k}}{\partial z_{k}^{t}}\\ \frac{\partial \gamma_{k}}{\partial x_{k}^{t}} & \frac{\partial \gamma_{k}}{\partial {\dot{x}}_{k}^{t}} & \frac{\partial \gamma_{k}}{\partial y_{k}^{t}} & \frac{\partial \gamma_{k}}{\partial {\dot{y}}_{k}^{t}} & \frac{\partial \gamma_{k}}{\partial z_{k}^{t}} \end{array}\right]\\ & = & \left[\begin{array}{ccccc} \frac{y_{k}^{r}}{x_{k}^{r^{2}}+y_{k}^{r^{2}}} & 0 & \frac{-x_{k}^{r}}{x_{k}^{r^{2}}+y_{k}^{r^{2}}} & 0 & 0\\ \frac{-x_{k}^{r}z_{k}^{r}}{\sqrt{x_{k}^{r^{2}}+y_{k}^{r^{2}}}\left(x_{k}^{r^{2}}+y_{k}^{r^{2}}+z_{k}^{r^{2}}\right)} & 0 & \frac{-y_{k}^{r}z_{k}^{r}}{\sqrt{x_{k}^{r^{2}}+y_{k}^{r^{2}}}\left(x_{k}^{r^{2}}+y_{k}^{r^{2}}+z_{k}^{r^{2}}\right)} & 0 & \frac{\sqrt{x_{k}^{r^{2}}+y_{k}^{r^{2}}}}{x_{k}^{r^{2}}+y_{k}^{r^{2}}+z_{k}^{r^{2}}} \end{array}\right] \end{array}$$

For track initialization at k=1, the Jacobian matrix of the angle-only measurements and the terrain height measurement can be formed as,

(A4) $$\begin{array}{ccc} H_{z} & = & \left[\begin{array}{ccccc} \frac{\partial \theta_{1}}{\partial x_{1}^{t}} & \frac{\partial \theta_{1}}{\partial {\dot{x}}_{1}^{t}} & \frac{\partial \theta_{1}}{\partial y_{1}^{t}} & \frac{\partial \theta_{1}}{\partial {\dot{y}}_{1}^{t}} & \frac{\partial \theta_{1}}{\partial z_{1}^{t}}\\ \frac{\partial \gamma_{1}}{\partial x_{1}^{t}} & \frac{\partial \gamma_{1}}{\partial {\dot{x}}_{1}^{t}} & \frac{\partial \gamma_{1}}{\partial y_{1}^{t}} & \frac{\partial \gamma_{1}}{\partial {\dot{y}}_{1}^{t}} & \frac{\partial \gamma_{1}}{\partial z_{1}^{t}}\\ \frac{\partial z_{1}^{t}}{\partial x_{1}^{t}} & \frac{\partial z_{1}^{t}}{\partial {\dot{x}}_{1}^{t}} & \frac{\partial z_{1}^{t}}{\partial y_{1}^{t}} & \frac{\partial z_{1}^{t}}{\partial {\dot{y}}_{1}^{t}} & \frac{\partial z_{1}^{t}}{\partial z_{1}^{t}} \end{array}\right]\\ & = & \left[\begin{array}{ccccc} \frac{y_{1}^{r}}{x_{1}^{r^{2}}+y_{1}^{r^{2}}} & 0 & \frac{-x_{1}^{r}}{x_{1}^{r^{2}}+y_{1}^{r^{2}}} & 0 & 0\\ \frac{-x_{1}^{r}z_{1}^{r}}{\sqrt{x_{1}^{r^{2}}+y_{1}^{r^{2}}}\left(x_{1}^{r^{2}}+y_{1}^{r^{2}}+z_{1}^{r^{2}}\right)} & 0 & \frac{-y_{1}^{r}z_{1}^{r}}{\sqrt{x_{1}^{r^{2}}+y_{1}^{r^{2}}}\left(x_{1}^{r^{2}}+y_{1}^{r^{2}}+z_{1}^{r^{2}}\right)} & 0 & \frac{\sqrt{x_{1}^{r^{2}}+y_{1}^{r^{2}}}}{x_{1}^{r^{2}}+y_{1}^{r^{2}}+z_{1}^{r^{2}}}\\ 0 & 0 & 0 & 0 & 1 \end{array}\right] \end{array}$$

To estimate the bias with unknown targets, the state vector is formulated as $x_{k}^{\mathrm{aug}2}={\left[x_{k}^{to},y_{k}^{to},z_{k}^{to},\theta_{b_{k}},\gamma_{b_{k}}\right]}^{T}$. Knowing the measurement vector $z_{k}=\left[\theta_{k},\gamma_{k}\right]$, partial derivatives with respect to the augmented state is shown below,

(A5) $$\begin{array}{ccc} H_{k}^{\mathrm{aug}2} & = & \left[\begin{array}{ccccc} \frac{\partial \theta_{k}}{\partial x_{k}^{to}} & \frac{\partial \theta_{k}}{\partial y_{k}^{to}} & \frac{\partial \theta_{k}}{\partial z_{k}^{to}} & \frac{\partial \theta_{k}}{\partial \theta_{b_{k}}} & \frac{\partial \theta_{k}}{\partial \gamma_{b_{k}}}\\ \frac{\partial \gamma_{k}}{\partial x_{k}^{to}} & \frac{\partial \gamma_{k}}{\partial y_{k}^{to}} & \frac{\partial \gamma_{k}}{\partial z_{k}^{to}} & \frac{\partial \gamma_{k}}{\partial \theta_{b_{k}}} & \frac{\partial \gamma_{k}}{\partial \gamma_{b_{k}}} \end{array}\right]\\ & = & \left[\begin{array}{ccccc} \frac{y_{k}^{rto}}{x_{k}^{{rto}^{2}}+y_{k}^{{rto}^{2}}} & \frac{-x_{k}^{rto}}{x_{k}^{{rto}^{2}}+y_{k}^{{rto}^{2}}} & 0 & 1 & 0\\ \frac{-x_{k}^{rto}z_{k}^{rto}}{\sqrt{x_{k}^{{rto}^{2}}+y_{k}^{{rto}^{2}}}\left(x_{k}^{{rto}^{2}}+y_{k}^{{rto}^{2}}+z_{k}^{{rto}^{2}}\right)} & \frac{-y_{k}^{rto}z_{k}^{rto}}{\sqrt{x_{k}^{{rto}^{2}}+y_{k}^{{rto}^{2}}}\left(x_{k}^{{rto}^{2}}+y_{k}^{{rto}^{2}}+z_{k}^{{rto}^{2}}\right)} & \frac{\sqrt{x_{k}^{{rto}^{2}}+y_{k}^{{rto}^{2}}}}{x_{k}^{{rto}^{2}}+y_{k}^{{rto}^{2}}+z_{k}^{{rto}^{2}}} & 0 & 1 \end{array}\right] \end{array}$$

For initialization, the Hessian matrix $H_{z}^{\mathrm{aug}2}$ is expressed as,

(A6) $$\begin{array}{ccc} H_{z}^{\mathrm{aug}2} & = & \left[\begin{array}{ccccc} \frac{\partial \theta_{1}}{\partial x_{1}^{to}} & \frac{\partial \theta_{1}}{\partial y_{1}^{to}} & \frac{\partial \theta_{1}}{\partial z_{1}^{to}} & \frac{\partial \theta_{1}}{\partial \theta_{b_{1}}} & \frac{\partial \theta_{1}}{\partial \gamma_{b_{1}}}\\ \frac{\partial \gamma_{1}}{\partial x_{1}^{to}} & \frac{\partial \gamma_{1}}{\partial y_{1}^{to}} & \frac{\partial \gamma_{1}}{\partial z_{1}^{to}} & \frac{\partial \gamma_{1}}{\partial \theta_{b_{1}}} & \frac{\partial \gamma_{1}}{\partial \gamma_{b_{1}}}\\ \frac{\partial z_{1}^{to}}{\partial x_{1}^{to}} & \frac{\partial z_{1}^{to}}{\partial y_{1}^{to}} & \frac{\partial z_{1}^{to}}{\partial z_{1}^{to}} & \frac{\partial z_{1}^{to}}{\partial \theta_{b_{1}}} & \frac{\partial z_{1}^{to}}{\partial \gamma_{b_{1}}} \end{array}\right]\\ & = & \left[\begin{array}{ccccc} \frac{y_{1}^{rto}}{x_{1}^{{rto}^{2}}+y_{1}^{{rto}^{2}}} & \frac{-x_{1}^{rto}}{x_{1}^{{rto}^{2}}+y_{1}^{{rto}^{2}}} & 0 & 1 & 0\\ \frac{-x_{1}^{rto}z_{1}^{rto}}{\sqrt{x_{1}^{{rto}^{2}}+y_{1}^{{rto}^{2}}}\left(x_{1}^{{rto}^{2}}+y_{1}^{{rto}^{2}}+z_{1}^{{rto}^{2}}\right)} & \frac{-y_{1}^{rto}z_{1}^{rto}}{\sqrt{x_{1}^{{rto}^{2}}+y_{1}^{{rto}^{2}}}\left(x_{1}^{{rto}^{2}}+y_{1}^{{rto}^{2}}+z_{1}^{{rto}^{2}}\right)} & \frac{\sqrt{x_{1}^{{rto}^{2}}+y_{1}^{{rto}^{2}}}}{x_{1}^{{rto}^{2}}+y_{1}^{{rto}^{2}}+z_{1}^{{rto}^{2}}} & 0 & 1\\ 0 & 0 & 1 & 0 & 0 \end{array}\right] \end{array}$$
